# Poor immune status and systemic disease are independently associated with mortality in AIDS-related Kaposi Sarcoma in Nigeria

**DOI:** 10.1186/1750-9378-7-S1-P7

**Published:** 2012-04-19

**Authors:** Patricia Agaba, Halima Sule, Raphael Ojoh, Zakary Saidu, Mohamed Mua’zu, Oche Agbaji, John Idoko, Ernest Ekong, Robert Murphy, Phyllis Kanki

**Affiliations:** 1 APIN Centre, Jos University Teaching Hospital, Jos, Nigeria; 2 AIDS Prevention Initiative Nigeria Plus, Abuja, Nigeria; 3 Northwestern University Medical School, Chicago, IL, USA; 4 Immunology and Infectious Diseases, Harvard School of Public Health, Boston, MA, USA

## Background

AIDS-related Kaposi’s sarcoma (AIDS-KS) is the most common AIDS-associated malignancy and remains a significant cause of morbidity and mortality in sub-Saharan Africa. We describe the determinants of mortality among patients with AIDS-KS in a comprehensive HIV care and treatment program in Jos, Nigeria.

## Material and methods

We collected epidemiologic, clinical, staging and survival data for 357 patients with a diagnosis of AIDS-KS enrolling for HIV-care at the Jos University Teaching Hospital. Patients were staged according to the AIDS Clinical Trials Group (ACTG) criteria, which are based on the evaluation of tumor extension (T), CD4+ cell count (I), and patient’s systemic status (S), stratified by good (0) versus poor (1) risk. Information on survival was obtained through an active follow-up on verification of vital status of the patients. Survival analysis was computed by the Kaplan-Meier method, and the log-rank test was used to test the difference between subgroups.

## Results

During the period of the study (2004-2008), there were 197 women (55.2%) and 160 (44.8%) men with AIDS-related Kaposi Sarcoma. Their mean age was 37±8 years and the median follow-up was 15 months (1-49 months). The median CD4+ and viral load were 107cells/mm³ and 58,561copies/ml respectively at baseline. Only 42 (11.8%) were on HAART at KS diagnosis, however all patients were commenced on HAART in line with existing national guidelines subsequently. 262(74.4%) had poor immune system status (I1: CD4+<200cells/mm^3^), 77.5% had widespread tumor extension (T1) and 80.2% had systemic disease (S1). Poor immune system (I1) status (AOR 2.07, CI 1.25-3.42, p=0.002) and presence of systemic disease (S1) (AOR 2.10, CI 1.03-4.28, p=0.004) were independently associated with mortality.

Regarding ACTG classification, the 4-year survival rate was 67% for I0 vs 46% for I1 (p=0.05), 58% for S0 vs 49% for S1 (p=0.41), 60% for T0 vs 46% for T1 (p=0.19).

## Conclusion

Poor immune status and systemic disease are independent predictors of mortality in patients with AIDS-KS in Nigeria.

